# Changes in the Prevalence of Overweight and Obesity among Peruvian Children under Five Years before and during the COVID-19 Pandemic: Findings from a Nationwide Population-Based Study

**DOI:** 10.3390/ijerph191912390

**Published:** 2022-09-29

**Authors:** Akram Hernández-Vásquez, Rodrigo Vargas-Fernández

**Affiliations:** 1 Centro de Excelencia en Investigaciones Económicas y Sociales en Salud, Vicerrectorado de Investigación, Universidad San Ignacio de Loyola, Lima 15024, Peru; 2 Facultad de Ciencias de la Salud, Universidad Científica del Sur, Lima 15067, Peru

**Keywords:** childhood overweight, childhood obesity, COVID-19, epidemiology, cross-sectional studies, Peru

## Abstract

This study aimed to identify changes in the prevalence of childhood (children under five years of age) overweight and obesity in Peru as a whole and at the departmental level, before and during the coronavirus disease (COVID-19) pandemic. We performed a secondary data analysis of two Demographic and Family Health Surveys (2019 and 2021) in Peru. The outcome was childhood overweight and obesity, defined as a weight-for-height score greater than 2 standard deviations. Poisson log generalized linear regression models adjusted for sex and/or age in months of the child were fitted to obtain the prevalence ratios of the changes in childhood overweight and obesity from 2019 to 2021. The analysis included 41,533 (2019: 20,414; 2021: 21,119) participants. The prevalence of childhood overweight and obesity was 6.4% in 2019 and 7.8% in 2021. Female children, aged 2, 3 and 4 years, and mothers who self-identified as non-native, had secondary and higher education, belonged to the middle and richer wealth quintile and resided in an urban area, in a village, in a small city and in the coastal region showed the largest increases in the prevalence of childhood overweight and obesity in 2021 compared to 2019. The departments of Pasco, Apurímac, Junín, Cusco, Lambayeque and La Libertad presented the largest increases in the prevalence of these nutritional disorders. During the pandemic, an increase in the prevalence of childhood overweight and obesity was observed, with demographic and socioeconomic factors accounting for the largest increases in the prevalence rates. A restructuring of overweight and obesity control strategies is required to curb this steady increase.

## 1. Introduction

Childhood overweight and obesity are a growing problem that has reached pandemic figures in the last two decades and have become major challenges for global public health [1]. Although in 2014 the World Health Organization (WHO) proposed that there be no increase in childhood overweight by 2025 [2], it is estimated that the global prevalence of overweight increased from 4.8% in 1990 to 5.9% in 2018, while obesity increased from 3.9% to 7.2% in boys and from 3.7% to 6.4% in girls during the period from 1980 to 2015 [1]. This increase in the prevalence of childhood overweight and obesity is associated with the onset of comorbidities during childhood such as psychological disorders, liver complications, musculoskeletal problems and metabolic and cardiovascular risk factors [3,4,5,6]. In addition, children with obesity are more likely to have comorbidities in adulthood [7], such as high blood pressure, dyslipidemia, type 2 diabetes and some obesity-related cancers. In this sense, the control of childhood overweight and obesity is a priority for health care systems worldwide due to the high long-term disease burden and high health care cost associated with these conditions [8].

The prevalence of childhood overweight and obesity varies between and within regions, with low- and middle-income countries encountering the greatest challenges in controlling these problems in the child population [9]. In Latin America and the Caribbean (LAC), one in five children and adolescents is overweight or obese, which places these regions among those with the highest rate of childhood obesity in the world and represents one of the greatest public health challenges in these areas [10]. Specifically, in Peru (one of the countries in LAC), the Nutritional Status Information System (SIEN is the acronym in Spanish) reported that the prevalence of overweight and obesity in children under five years of age recorded in public health facilities increased from 6.1% and 1.5% in 2016 to 6.9% and 2.2% in 2021 [11], reflecting a steady increase in these nutritional problems in the child population. Based on previous studies [12,13,14], weight gain has a multifactorial etiopathogenesis based on individual, familial, social, behavioral and genetic factors that interact with each other, generating high levels of childhood overweight and obesity. However, these obesogenic factors may have been modified by the Coronavirus disease pandemic (COVID-19) due to the social, economic, health and behavioral changes caused by this disease worldwide.

Currently, the indirect impact of the COVID-19 pandemic has led to negative lifestyle changes in people (including children and adolescents), which has rapidly increased the rate of childhood obesity worldwide [15,16]. Particularly, preventive measures instituted by governments around the world, such as social confinement, physical distancing, closure of schools and recreational areas, caused a change in physical activity, diet, and sleep behaviors in children and adolescents during the pandemic period [17]. According to the biomedical literature, school or daycare closures and exposure to out-of-school environments favor obesogenic behaviors causing weight gain in children and adolescents, as opposed to the school period [18]. Likewise, social confinement and the closure of recreational environments generated unstructured periods (as opposed to school routine), in which children were less active (a study in the United States found that children remained seated 91.1 min for school activities, and 398.5 min for leisure activities) [19], consumed more foods high in saturated fat, sugar and salt (in a Canadian study, 42% of children consumed more food, and 55% consumed more snacks) [20], spent prolonged screen time [21] and had irregular sleep patterns [22]. This exacerbation of obesogenic behaviors during the pandemic predisposed children to weight gain, which would have a negative impact on their physical and mental well-being.

In Peru, information is only available on the prevalence of childhood overweight and obesity before and during the COVID-19 pandemic in children under five years of age who attended public health facilities [11], while the Demographic and Family Health Survey (ENDES is the acronym in Spanish) does not report the prevalence of these nutritional problems in the national territory [23,24]. Therefore, the objective of the present study was to identify changes in the prevalence of childhood (under five years of age) overweight and obesity in Peru and at the departmental level, before and during the COVID-19 pandemic.

## 2. Materials and Methods

### 2.1. Data Source and Data Description

The data used for this study were obtained from the ENDES, which is representative of the whole nation and collects information on reproductive health, maternal and child health and noncommunicable diseases [25,26]. For our analysis, we included data from the 2019 and 2021 ENDES in order to compare the COVID-19 pre-pandemic scenario (year 2019) with the last available year (2021) during the pandemic [25,26].

The ENDES is implemented by the National Institute of Statistics and Informatics (INEI is the acronym in Spanish) and collects information through three questionnaires: the Household Questionnaire, the Individual Questionnaire for Women and the Health Questionnaire. The Individual Questionnaire for Women is addressed to women aged 12 to 49 years, and its main objective is to provide information on the health status of mothers and children under five years of age [25,26]. The sampling framework consists of the statistical and cartographic information from the National Censuses and the cartographic material produced for the execution of the 2019 and 2021 ENDES [25,26].

### 2.2. Sampling and Data Collection

**Figure 1:**
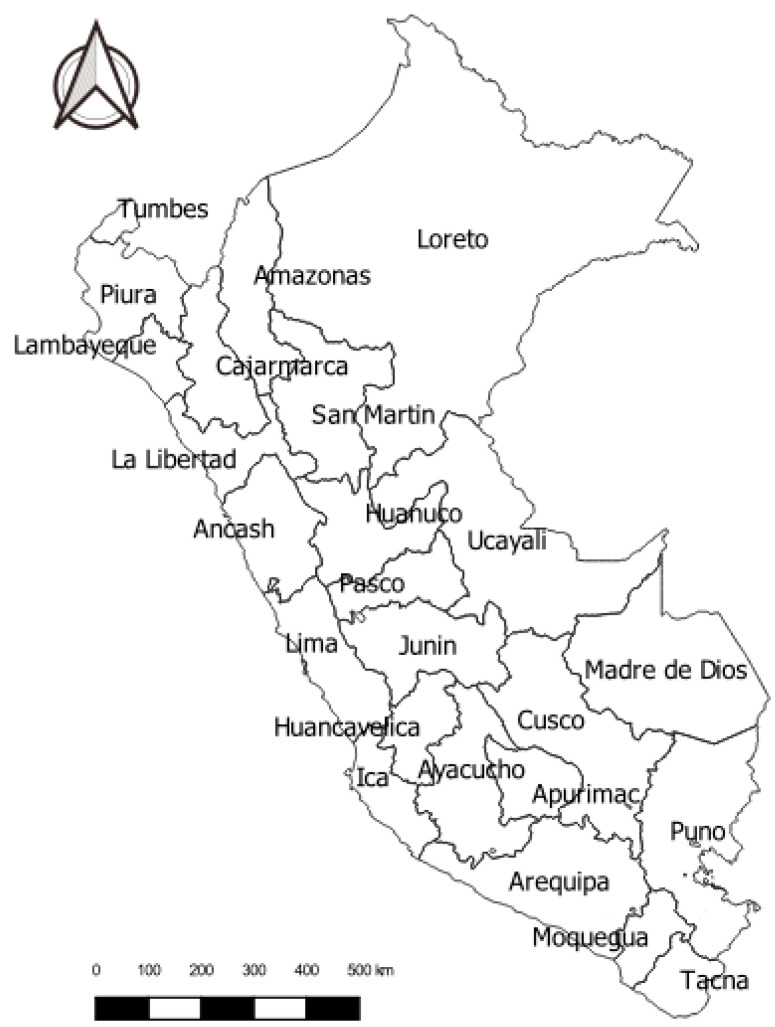
Map of the departments of Peru according to a political-administrative division (own elaboration using QGIS).

The ENDES sample is two-stage, probabilistic, balanced, stratified and independent, carried out at the departmental level (according to the political-administrative division of the Peruvian territory in Figure 1) and in urban and rural areas [25,26]. The sampling units in urban areas are conglomerates of contiguous dwellings and private dwellings, whereas in rural areas, they are rural census areas and private dwellings. The survey’s research unit consists of the usual residents of private dwellings in urban and rural areas of the country who have spent the night before the survey in the selected dwelling. The collection of coverage information in the selected dwellings is carried out through a mobile device (Tablet). The method used involves direct interviews conducted by personnel duly trained to collect the requested information [25,26]. Further details on the sampling process and the design of the 2019 and 2021 ENDES can be found in the respective data sheets [25,26].

**Figure 2:**
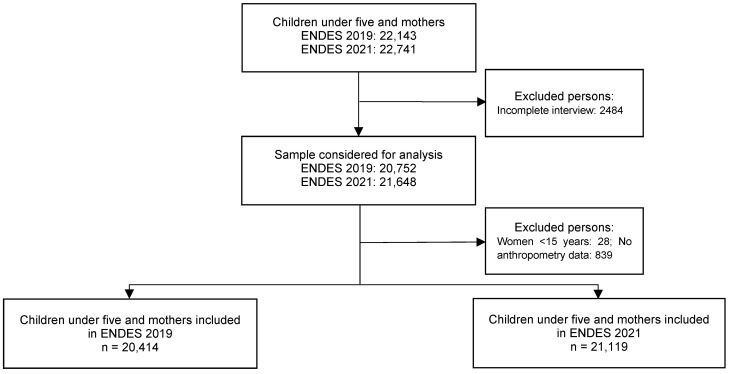
Flowchart of the selection of children under 5 years of age and mothers included in the study.

The ENDES reports anthropometric measurements (weight and height) of women aged 12 to 49 years and children under 5 years of age listed in the Household Questionnaire (usual residents and visitors); children with Down syndrome are not included [27,28]. Women aged 15 to 49 years and children under 5 years with complete data on weight, height and variables of interest were included in this study. The final sample for the analysis consisted of 20,414 (year 2019) and 21,119 (year 2021) women aged 15–49 years and children under 5 years who were usual household residents. The flow diagram of the study is presented in Figure 2.

### 2.3. Variables

In this study, the outcome was childhood overweight and obesity defined as a weight-for-height score greater than 2 standard deviations (SD) (taking value 1) according to the reference standards established by the WHO, included in the Table of anthropometric nutritional assessment of children under 5 years of the Ministry of Health of Peru [29,30]. The work teams were composed of two interviewers, an anthropometrist and a supervisor who performed the anthropometric measurements in the selected households. For the height measurements, a multi-purpose infantometer or a mobile measuring rod was used, which underwent a quality control before each measurement and had an accuracy of less than or equal to 2 millimeters. For the weight measurement, an electronic foot scale (Seca models 872, 874, 878) was used. Height was recorded in centimeters, with millimeters to one decimal place, and weight was recorded in kilograms, with grams to one decimal place. In the case of children under 2 years of age, their length was measured while lying down, while the height of children who were 2 years old or older was measured while the child was standing. For weight, in the case of children under 2 years of age, we measured first the weight of the mother and then the weight of the mother with the child in her arms, establishing the difference between the measurements as the child’s weight. If the child was 2 years old or older, the weight was measured while the child was standing on a scale. Further details of the process of anthropometric measurements can be found in the Manuals of the Interviewer of the 2019 and 2021 ENDES [27,28,31,32].

Other variables included for the stratification of the results or adjustments were the sex of the child (male, female), the age of the child in years (0, 1, 2, 3, 4 years), the age of the child in months (0 to 59 months), the age of the mother (15–19, 20–34, 35–49 years), the ethnicity (non-native, native), the educational level (up to primary, secondary education, higher education), the wealth quintile (poorest, poorer, middle, richer, richest), the area of residence (urban, rural), the place of residence (countryside, town, small city, capital), and the natural area of residence (coast, highlands, jungle).

### 2.4. Statistical Analysis

Data analysis included descriptive and inferential analyses including complex sampling characteristics and sampling weights for each of the ENDES years. Descriptive analysis was used to report the frequency distribution of the mothers’ and children’s characteristics and the prevalence rates with their 95% confidence intervals for childhood overweight and obesity by survey years. Poisson log generalized linear regression models adjusted for covariates (sex and/or age in months of the child) were fitted to obtain the prevalence ratios of the change in childhood overweight and obesity from 2019 to 2021, with the outcome being childhood overweight and obesity, and the independent variable the survey year according to the variables of interest. Likewise, prevalence rates and percentage changes in the departmental prevalence values of childhood overweight and obesity between 2019 and 2021 were calculated.

The level of statistical significance was 5%. All statistical analyses were performed in Stata 17 (StataCorp, College Station, TX, USA). Python version 3.8 programming language was used to plot the prevalence rates and the percentage changes in the departmental prevalence values of childhood overweight and obesity. The graph code is publicly available on GitHub (https://github.com/ahernandezv/Plot_Childhood_Overweight_Obesity).

### 2.5. Ethical Considerations

Ethical review and approval were waived for this study, since all the data from the ENDES are publicly accessible on the INEI website: http://iinei.inei.gob.pe/microdatos/ (accessed on 10 June 2022).

## 3. Results

A total of 41,533 (2019: 20,414; 2021: 21,119) children under 5 years of age and their mothers were included in the analysis. Regarding the demographic characteristics of the 2019 and 2021 samples, we found that most children were 3 years old (2019: 20.7%; 2021: 21.6%) and male (2019: 50.7%; 2021: 50.5%), while most of the mothers were between 20 and 34 years old (2019: 66.2%; 2021: 65.1%), self-identified as non-native (2019: 93.3%; 2021: 94.0%), had secondary as their highest educational level (2019: 46.0%; 2021: 47.1%), belonged to the poorest wealth quintile (2019: 25.5%; 2021: 25.4%), and resided in an urban area (2019: 72.7%; 2021: 73.7%) and in the coastal region (2019: 53.9%; 2021: 54.3%). On the other hand, it was found that in 2019, most of the mothers resided in the countryside (27.3%) and in the capital (27.3%), while in 2021, the majority resided in the countryside (26.3%) and in a town (26.6%) (Table 1).

The prevalence of childhood overweight and obesity adjusted for age and/or sex was 6.4% in 2019 and 7.8% in 2021 (21% increase from 2019 to 2021). Regarding the proportions of childhood overweight and obesity according to sociodemographic characteristics, it was found that the majority of the children were female (2019: 6.6%; 2021: 8.5%) and were less than 1 year old (2019: 12.6%; 2021: 11.2%), and the majority of their mothers self-identified as non-native (2019: 6.7%; 2021: 8.1%), belonged to the richest wealth quintile (2019: 11.2%; 2021: 12.6%) and resided in an urban area (2019: 7.5%; 2021: 9.4%), in the capital (2019: 10.3%; 2021: 12.2%) and in the coastal region (2019: 9.0%; 2021: 11.2%). However, it was found that 6.7% of the mothers were between 35 and 49 years old in 2019, while 7.9% of the mothers were between 20 and 34 years old in 2021 (Table 2).

Regarding the comparison of the prevalence of childhood overweight and obesity adjusted for age and/or sex according to sociodemographic characteristics, it was found that, compared to 2019, the largest increases in the prevalence of childhood overweight and obesity in 2021 were reported in female children (+29%), who were 2 (+59%), 3 (+34%) and 4 years old (+45%) and whose mothers were between 20 and 34 years old (+26%), self-identified as non-native (+21%), had secondary (+18%) and higher (+31%) education, belonged to the middle (+39%) and richer (+39%) wealth quintile and resided in an urban area (+25%), in a town (+20%), in a small city (+52%) and in the coastal region (+24%) (Table 2).

**Figure 3:**
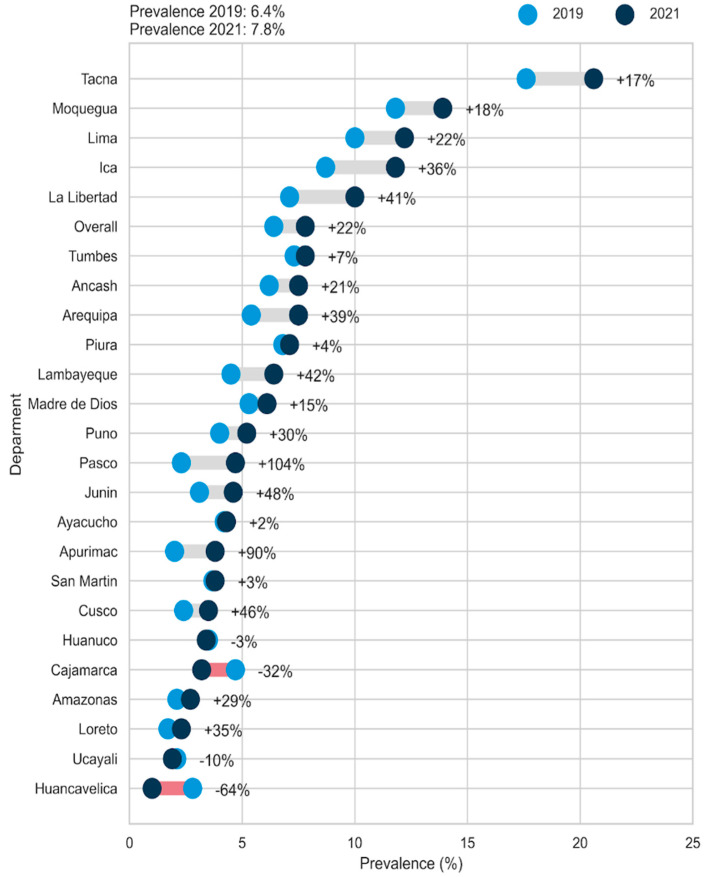
Comparison of the departmental prevalence rates of childhood overweight and obesity, 2019 and 2021 ENDES.

In relation to the departmental prevalence rates (the 24 departments of Peru were included, with the Constitutional Province of Callao being considered within the department of Lima), the departments of Pasco (+104%), Apurímac (+90%), Junín (+48%), Cusco (+46%), Lambayeque (+42%) and La Libertad (+41%) presented the largest increases in the prevalence of childhood overweight and obesity, while the departments of Huancavelica (−64%), Cajamarca (−32%), Ucayali (−10%) and Huánuco (−3%) presented reductions in the prevalence of these disorders. In addition, the highest prevalence rates were found in Tacna, Moquegua, Lima, and Ica (Figure 3).

## 4. Discussion

The present study aimed to identify changes in the prevalence of childhood overweight and obesity in Peru as a whole and at the departmental level, before and during the COVID-19 pandemic. Overall, a 21% increase in the prevalence of childhood overweight and obesity was found between 2019 and 2021. In addition, the largest increases in the prevalence of overweight and obesity in 2021 compared to 2019 were found in female children, aged 2, 3 and 4 years, with mothers who self-identified as non-native, had secondary and higher education, belonged to the middle and richer wealth quintile and resided in an urban area, in a village, in a small city and in the coastal region. At the departmental level, the largest increases in the prevalence of overweight and obesity were found in the departments of Pasco, Apurímac, Junín, Cusco, Lambayeque, and La Libertad, while the departments of Huancavelica, Cajamarca, Ucayali and Huánuco presented reductions in the prevalence of these disorders in 2021 compared to 2019. In addition, the highest prevalence rates were found in Tacna, Moquegua, Lima and Ica.

The prevalence of childhood overweight and obesity was found to be 6.4% in 2019 and 7.8% in 2021. This finding is similar to those reported in a systematic review (which evaluated the impact of confinement on body weight in the pediatric population) [33] and in studies conducted in Israel [34], the United States [35], England [36] and China [37,38], in which an increase was found in the prevalence of overweight and obesity as measured by weight gain and standardized body mass index. Our finding can be attributed to changes in people’s lifestyles brought about by pandemic control strategies to decrease the spread of the SARS-CoV-2 virus; in particular, a reduction in physical activity, changes in eating habits, increased sedentary behaviors and school closures were the main factors contributing to the recorded weight gain in children and adolescents [36,39,40,41]. With respect to physical activity, the biomedical literature reports that confinement, school closures and restrictions on the access to outdoor recreational venues during the COVID-19 pandemic resulted in children experiencing a disordered daily routine and schedule (compared to the school period) and adopting sedentary behaviors related to a decreased duration and frequency of physical activity and an increased time spent in front of an electronic device for educational and leisure activities, which contributed to an increase in total and abdominal fat or insulin resistance levels in children [19,42,43,44]. On the other hand, during the pandemic, changes in the eating habits characterized by an increase in the consumption of high-calorie processed foods (sugary drinks, saturated fats and salt) occurred in children due to the low cost of this type of food and beverages and to a decreased parental control over their children’s diets [39,45,46]. Moreover, movement restrictions due to social confinement and food insecurity during the pandemic in various regions of the world such as Latin America [47,48] limited the access to fresh and unprocessed foods such as fruits and vegetables, contributing to an inadequate nutrition of the children and, consequently, to an increase in childhood overweight and obesity [49].

Another factor that could promote weight gain is a change in sleeping habits in children in various regions of the world during the COVID-19 pandemic [22,50]. According to the biomedical literature, the preventive measures put in place by governments during the COVID-19 pandemic resulted in delayed bedtime and longer periods of wakefulness due to the increased use of electronic devices and a poorly structured routine, which resembled the sleep patterns observed during vacation periods [22,50]. This sleep–wake disturbance during the social confinement led to increased food consumption during the night, altered hormonal circadian cycle and increased sleeping time, which would reduce the frequency of physical activity [51,52,53]. On the other hand, the COVID-19 pandemic introduced periods of chronic stress in the parents due to unemployment, social isolation, changes in family dynamics and constant fear about SARS-CoV-2 infection, which had an impact on the children, causing high levels of stress, fear and anxiety [54,55]. Prolonged stress experienced by children could lead to weight gain due to the stimulation of chronic cortisol secretion and could lead to a depressive disorder associated with isolation, staying at home, sedentary behavior, and unhealthy eating [17,56,57]. Additionally, parents who experience periods of chronic stress and lack of social support have an inability to control and regulate their children’s eating behavior, physical activity and screen time, which also contributes to children’s weight gain [58]. In this sense, childhood obesity treatment programs (especially, lifestyle interventions) should focus on face-to-face or virtual counseling of the parents or caregivers on the main changes brought about by the preventive measures implemented during the COVID-19 pandemic, to control the obesogenic factors experienced by the children and decrease the prevalence of childhood overweight and obesity.

On the other hand, it was found that female children, aged 2, 3 and 4 years, and mothers who self-identified as non-native, had secondary and higher education, belonged to the middle and richer wealth quintile, and resided in an urban area, in a village, in a small city and in the coastal region reported the highest increases in the prevalence of overweight and obesity in 2021. Childhood overweight and obesity could be due to biological, social, and environmental factors that predispose to weight gain in children [59]. In general, studies conducted in the pre-pandemic years reported that the highest prevalence of childhood overweight and obesity was found in mothers with secondary and higher education, belonging to a richer and richest wealth quintile and residing in an urban setting [14,60]. Regarding maternal education, the biomedical literature reports that a higher maternal educational level is associated with a higher consumption of animal proteins and fats, less physical activity and increased sedentary behavior, which could have an impact on the weight gain of children [61,62]. Likewise, having a high socioeconomic level and residing in an urban area are social determinants that contribute to an excessive weight in children [63]. Regarding the socioeconomic status, the availability and access to food would contribute to the consumption of foods with a high caloric content and low nutritional value [64], while residing in an urban environment is associated with changes in eating habits (higher consumption of processed and high-calorie foods) and lower physical activity [65].

Likewise, studies conducted in the pre-pandemic years showed that children older than 2 years of age experience an increase in protein intake through the intake of complementary foods to breastfeeding, and it is postulated that the protein intake would be three times higher than the physiological requirement, contributing to an increase in adiposity that leads to high levels of childhood overweight and obesity [66]. Mothers’ ethnicity could influence the prevalence of these nutritional disorders because cultural practices predispose to greater physical activity related to domestic work and agriculture, lower socioeconomic income, lower commercialization of high-calorie foods and higher prevalence of malnutrition in the homes of native mothers [67,68]. On the other hand, it was found that female children reported higher increases in the prevalence of overweight and obesity, which differs from the biomedical literature of the pre-pandemic years, when males were found to possess biological and sociocultural characteristics that contributed to a higher prevalence of overweight and obesity compared to females [69]. However, the closure of schools during the COVID-19 pandemic could have led to a decrease in physical activity in both sexes, possibly contributing to an increase in the prevalence of these nutritional disorders regardless of the sex of the children [70]. Therefore, considering the persistence of these factors during the pandemic, governmental institutions should establish strategies linked to the nutritional status of children, taking into account the biological, social and environmental factors that cause overweight in this population.

Regarding the departmental prevalence rates, the greatest increases were found in the departments of Pasco, Apurímac, Junín, Cusco, Lambayeque and La Libertad, while the departments of Huancavelica, Cajamarca, Ucayali and Huánuco presented reductions in the prevalence of childhood overweight and obesity. In addition, the highest prevalence rates were found in Tacna, Moquegua, Lima and Ica. Previous studies reported that the highest prevalence rates of childhood overweight and obesity in the pre-pandemic years were recorded in departments located in the coastal region of Peru, such as Lima, Ica, Moquegua and Tacna, while departments with a lower prevalence of overweight and obesity were located in the highlands and jungle region, such as Loreto, Junín, Cusco, San Martín, Apurímac and Ayacucho [71,72]. Based on our findings, the impact of the pandemic generated the largest increases in the prevalence of childhood overweight and obesity in the departments located in the highlands and jungle region (Pasco, Apurímac, Junín and Cusco). This problem could lead to a steady increase in these nutritional disorders due to the socioeconomic characteristics observed in these regions, such as low socioeconomic status, limited access to nutritional health services, low agricultural production due to the context of the pandemic and less social support [73,74]. Particularly, Pasco and Apurimac had, before the pandemic, the highest proportions of residents living in poverty and extreme poverty [75], which could have worsened with the arrival of COVID-19 and indirectly impact on the food security and nutritional indicators. On the other hand, Tacna, Moquegua, Lima and Ica continue to have the highest prevalence of childhood overweight and obesity, which may be attributed to low physical activity levels and increased consumption of high-energy foods that may have been accentuated during the pandemic [76]. In this sense, government institutions should strengthen strategies for the control of childhood overweight and obesity, especially in the regions of the highlands and jungle, where the greatest socioeconomic and health inequalities are observed in the Peruvian territory and the prevalence of childhood overweight and obesity has increased the most.

Our findings could contribute to the implementation of public health measures. First, excess weight during childhood can remain into adulthood, increasing the morbidity and mortality in this age group, especially considering that obesity is a severity risk factor for COVID-19 [77]. Therefore, strategies to control overweight and obesity that are oriented to the first stage of life should be strengthened. Second, lifestyle interventions, such as a treatment for childhood obesity, should be implemented throughout the national territory, considering the economic and socio-cultural changes caused by the COVID-19 pandemic. Third, nutritional health services should focus on the departments showing the greatest increases in childhood overweight and obesity (located in the highlands and jungle regions) along with the departments of the coastal region that reported the highest prevalence rates. Finally, with the current reopening of the educational institutions, there could be an increase in the exposure to and consumption of foods with low nutritional content [78]; therefore, regulatory measures should be established to control the sale of processed and ultra-processed foods in school environments, promote healthy lifestyles and counteract the increase in the prevalence of childhood overweight and obesity.

The present study is not without limitations. In our study, the results were adjusted for sex and age, but it would be interesting to adjust for other variables such as physical activity level, dietary habits, sleep patterns and some variables related to the COVID-19 pandemic. In addition, the surveyors may have recorded inaccurate data regarding the height and weight of the children. Additionally, the 2019 and 2021 ENDES had different sample frames, which could compromise the comparability between the surveys; however, both surveys are representative of the Peruvian population. Nonetheless, the ENDES is the only survey conducted annually that is representative at the national, urban and rural levels as well as at the departmental level to evaluate nutritional indicators in children under 5 years of age and is conducted by personnel previously trained to collect information and maintain data quality.

## 5. Conclusions

In conclusion, the prevalence of childhood overweight and obesity increased by more than 1 percentage point between the pre-pandemic and the pandemic periods. The greatest increases in the prevalence rates were found in female children, aged 2 to 4 years, whose mothers self-identified as non-native, had secondary and higher education, belonged to the middle and richer wealth quintile and resided in an urban area, in a town, in a small city and in the coastal region. In addition, the departments of Pasco, Apurímac, Junín, Cusco, Lambayeque and La Libertad registered the largest increases at the departmental level. Therefore, strategies to control childhood overweight and obesity should be put into place, considering the economic and socio-cultural changes generated by the COVID-19 pandemic.

## Figures and Tables

**Table 1:** Summary statistics of children under 5 years of age and their mothers included in the study.

	2019 ENDES		2021 ENDES	
Characteristics	Sample	Percentage	Sample	Percentage
Overall	20,414	100	21,119	100
Sex of child				
Male	10,454	50.7	10,675	50.5
Female	9960	49.3	10,444	49.5
Age of child				
0 years	3682	17.6	3808	18.2
1 years	4097	20.2	4242	20.2
2 years	4121	20.3	4229	19.6
3 years	4219	20.7	4609	21.6
4 years	4295	21.2	4231	20.3
Mother age group				
15–19 years	975	4.6	878	4.1
20–34 years	13,644	66.2	13,852	65.1
35–49 years	5795	29.3	6389	30.8
Ethnicity				
Non-native	18,599	93.3	19,243	94.0
Native	1815	6.7	1876	6.0
Education level				
Up to primary	4089	20.3	4077	18.4
Secondary education	9548	46.0	10,148	47.1
Higher education	6777	33.7	6894	34.5
Wealth quintile				
Poorest	5613	25.5	6426	25.4
Poorer	5595	24.7	5595	23.8
Middle	4096	19.8	4113	20.6
Richer	3013	16.6	3069	17.4
Richest	2097	13.5	1916	12.8
Area of residence				
Urban	14,540	72.7	14,355	73.7
Rural	5874	27.3	6764	26.3
Place of residence				
Countryside	5874	27.3	6764	26.3
Town	5876	25.1	5841	26.6
Small city	6113	20.2	5895	21.0
Capital	2551	27.3	2619	26.1
Natural area				
Coast	8711	53.9	8805	54.3
Highlands	6616	28.0	6971	27.7
Jungle	5087	18.1	5343	18.0

ENDES: Demographic and Family Health Survey. All estimates took into account the ENDES sample design and weights. The place of residence is classified as capital (capital cities and cities with more than 1 million inhabitants), small city (more than 50,000 inhabitants) and town (other urban areas); countryside corresponds to rural areas.

**Table 2:** Summary statistics of children under 5 years of age and their mothers included in the study.

	Prevalence of Overweight/Obesity		
	ENDES 2019	ENDES 2021	2021 with Respect to 2019
Characteristics	% (95% CI)	% (95% CI)	PR (95% CI)
Overall	6.4 (6.0–6.9)	7.8 (7.3–8.3)	**1.21 (1.10–1.34)**
Sex of child *			
Male	6.2 (5.6–6.9)	7.1 (6.4–7.8)	1.14 (0.99–1.31)
Female	6.6 (5.9–7.3)	8.5 (7.8–9.2)	**1.29 (1.13–1.47)**
Age of child			
0 years	12.6 (11.2–14.1)	11.2 (10.0–12.6)	0.92 (0.78–1.08)
1 years	5.0 (4.1–6.0)	6.3 (5.4–7.4)	1.26 (0.99–1.62)
2 years	2.6 (2.0–3.3)	4.1 (3.3–5.0)	**1.59 (1.16–2.19)**
3 years	5.0 (4.2–6.0)	6.7 (5.8–7.7)	**1.34 (1.07–1.67)**
4 years	7.6 (6.6–8.8)	10.9 (9.7–12.2)	**1.45 (1.21–1.74)**
Mother age group			
15–19 years	6.6 (4.8–9.0)	5.5 (3.9–7.8)	0.86 (0.54–1.35)
20–34 years	6.3 (5.7–6.9)	7.9 (7.3–8.6)	**1.26 (1.12–1.42)**
35–49 years	6.7 (5.8–7.7)	7.7 (6.9–8.6)	1.16 (0.97–1.39)
Ethnicity			
Non-native	6.7 (6.2–7.2)	8.1 (7.5–8.6)	**1.21 (1.09–1.33)**
Native	2.6 (1.8–3.6)	3.3 (2.4–4.5)	1.30 (0.82–2.06)
Education level			
Up to primary	4.1 (3.3–5.0)	3.6 (3.0–4.4)	0.87 (0.66–1.16)
Secondary education	6.0 (5.4–6.8)	7.2 (6.5–7.9)	**1.18 (1.02–1.37)**
Higher education	8.3 (7.5–9.2)	10.9 (9.9–11.9)	**1.31 (1.14–1.50)**
Wealth quintile			
Poorest	3.0 (2.5–3.6)	2.8 (2.4–3.4)	0.95 (0.74–1.23)
Poorer	5.6 (4.8–6.5)	6.1 (5.3–7.1)	1.08 (0.88–1.33)
Middle	7.1 (6.1–8.2)	9.9 (8.7–11.2)	**1.39 (1.15–1.69)**
Richer	8.1 (6.8–9.5)	11.2 (9.8–12.8)	**1.39 (1.12–1.71)**
Richest	11.2 (9.5–13.2)	12.6 (10.9–14.5)	1.12 (0.90–1.39)
Area of residence			
Urban	7.5 (6.9–8.1)	9.4 (8.7–10.1)	**1.25 (1.12–1.39)**
Rural	3.4 (2.9–4.1)	3.3 (2.8–3.8)	0.96 (0.76–1.22)
Place of residence			
Countryside	3.4 (2.9–4.1)	3.3 (2.8–3.8)	0.96 (0.76–1.22)
Town	6.1 (5.4–7.0)	7.4 (6.6–8.2)	**1.20 (1.02–1.42)**
Small city	5.6 (4.9–6.3)	8.4 (7.5–9.5)	**1.52 (1.28–1.81)**
Capital	10.3 (9.0–11.6)	12.2 (10.8–13.7)	1.19 (1.00–1.42)
Natural area			
Coast	9.0 (8.2–9.8)	11.2 (10.3–12.1)	**1.24 (1.10–1.39)**
Highlands	3.7 (3.2–4.2)	4.0 (3.5–4.6)	1.11 (0.91–1.34)
Jungle	2.9 (2.4–3.5)	3.3 (2.8–4.0)	1.15 (0.89–1.50)

ENDES: Demographic and Family Health Survey, PR: Prevalence ratio, 95% CI: 95% confidence interval. All estimates took into account the ENDES sample design and were adjusted for age in months and sex of the child. The bold values denote statistical significance at the *p* < 0.05 level. * Adjusted only for age in months. The place of residence was classified as capital (capital cities and cities with more than 1 million inhabitants), small city (more than 50,000 inhabitants) and town (other urban areas); countryside corresponds to rural areas.

## Data Availability

The data from the ENDES are publicly accessible on the INEI website: http://iinei.inei.gob.pe/microdatos/ (accessed on 10 June 2022).
